# Patent Rights

**Published:** 1891-04

**Authors:** 


### Patent Rights.

The word "patent" means "open;" and it was first made use of to describe the open letters, or "letters patent," by which a sovereign granted to a citizen or subject a monopoly of the exclusive use or sale of any article.

The word is now, in a sense not originally intended, applied to the secret of an inventor which has by him been made open to the whole public in consideration of a right for a limited period to make and sell the thing invented.

The original patent was often for a monopoly of a commodity which the people already freely possessed. The king of England for instance, granted to individual subjects the exclusive right to sell such articles as salt and vinegar.

Such monopolies as this have been declared wrongful in England and have never existed in the United States. The only patentable thing with us is a useful invention or discovery which has never before been possessed by the people.

The theory of the patent right is this: The inventor possesses a secret likely to be useful to the people, which secret he reveals freely to the government, for the benefit of the general public. In return for his sevice in revealing his secret, the government grants him the exclusive right, for a limited time, to make and sell the thing which his invention covers.

The patent, therefore, is simply a contract between the inventor and the people, in which the latter grant the former the sole right for a time to the profits from this invention, in consideration of having the free possession of the invention later on.

In this country a patent lasts only seventeen years. After that time all are free to make full use of the patented invention without payment of any fee or royalty—unless, indeed, the patent is extended by special act of congress. This extension is rarely granted, and only in cases where it is held that the patentee has failed to get the benefit of his patent.

The term of a patent was formerly fourteen years, and it was renewable by the patent office for seven years more. This privilege of extension by the patent office was abolished in 1861, and the term made seventeen years.

There is much misapprehension of the ext^nt and character or the privilege which a patent really grants.

The government, in giving a patentee the right to the exclusive possession of his invention, does not undertake to guarantee him in that right. The invention is, during the term of his patent, his property; but he must defend it before the law in the same way that any other property must be defended.

The possessor of a horse, for instance, even if he has bought and paid for the animal, can not keep possession of it if some other person can prove that it belongs to him. The holder of a patent may lose his right to it in several ways; and if he does lose the right to it, those who have bought of him his commodity have no more right to use it further, or to be indemnified by the government for the loss of it or its use than the innocent holder of a stolen horse has to be protected in its possession.

It is possible that some other person may prove that he was in advance of the patentee in the possession or use of what the latter has claimed as his secret or invention. The patentee may have used fraud in obtaining his patent; or he may be selling, as his patented article, something which is not exactly what his application described.

If the government should undertake to guarantee to a patentee, against all comers, the exclusive right to and possession of the thing patented, it would be granting a wrongful monopoly, because this would be a denial of the equal right of all citizens to sue and be sued, and to obtain judgments in accordance with equity and right.

A thing already freely made and used can not be patented. The right to use it is common to all the people. And on the other hand, the liability of the purchaser of a patented article to lose the right to use it, provided it is proved to infringe some other person's rightful patent, is a part of the defence of the equal rights of all.—*Youth's Companion.*

